# Do You See What I See? Effectiveness of 360-Degree vs. 2D Video Ads Using a Neuroscience Approach

**DOI:** 10.3389/fpsyg.2021.612717

**Published:** 2021-02-19

**Authors:** Jose M. Ausin-Azofra, Enrique Bigne, Carla Ruiz, Javier Marín-Morales, Jaime Guixeres, Mariano Alcañiz

**Affiliations:** ^1^Instituto de Investigación e Innovación en bioingeniería (I3B), Universitat Politécnica de Valencia, Valencia, Spain; ^2^Departamento de Comercialización e Investigación de Mercados, Universidad de Valencia, Valencia, Spain

**Keywords:** 360-degree advertisement, effectiveness, electroencephalography, eye tracking, facial-coding, electrodermal activity, consumer neuroscience

## Abstract

This study compares cognitive and emotional responses to 360-degree vs. static (2D) videos in terms of visual attention, brand recognition, engagement of the prefrontal cortex, and emotions. Hypotheses are proposed based on the interactivity literature, cognitive overload, advertising response model and motivation, opportunity, and ability theoretical frameworks, and tested using neurophysiological tools: electroencephalography, eye-tracking, electrodermal activity, and facial coding. The results revealed that gaze view depends on ad content, visual attention paid being lower in 360-degree FMCG ads than in 2D ads. Brand logo recognition is lower in 360-degree ads than in 2D video ads. Overall, 360-degree ads for durable products increase positive emotions, which carries the risk of non-exposure to some of the ad content. In testing four ads for durable goods and fast-moving consumer goods (FMCG) this research explains the mechanism through which 360-degree video ads outperform standard versions.

## Introduction

Online video ad spend is growing exponentially (Zenith, 2019) and the watching time of online platforms such as YouTube is increasing (Google, 2019). Among the viewing technologies employed, 360-degree videos are widely used by realtors/real estate agents, tourism suppliers, and manufacturers across multiple product categories. Indeed, large companies such as Disney, McDonalds, Coca-Cola, BMW, and Audi have conducted 360-degree video-based campaigns. The use of 360-degree videos is one of the newest trends in online marketing (Gudacker, 2016; Castellanos et al., 2018; Feng et al., 2019). Due to their interactive nature, rich imagery, and spatial sound design, 360-degree videos have been termed “the ultimate empathy machine” (Feng et al., 2019).

A 360-degree video is a monoscopic video viewed as one image directed to both eyes. It provides free and omnidirectional viewpoints from all the angles of a 360-degree radius under the control of the viewer. The use of 360-degree ads has highlighted two complementary features of research interest. First, the change from static to an interactive view provides a new customer experience. Due to its interactivity, the 360-degree video seems to provide a stronger connection than does traditional video with the human mind and increases users' immersive settings (Sundar et al., 2014). Second, when users control the angle of view they may feel a sense of active viewing, which demands higher cognitive effort. Moreover, the higher cognitive load associated with 360-degree videos might result in different levels of brand memory and persuasion in comparison with 2D videos. Although 360-degree videos give viewers a higher degree of immersion than do 2D formats, and a significant level of control, that shapes their viewing experiences, the viewers may become confused and not enjoy the experience as they try to figure out the “correct” viewing path (Feng, 2018). Therefore, the efficacy of 360-degree video ads should be examined (Feng, 2018). Surprisingly, despite the growth in the use of 360-degree videos, and their distinctive features, there has been little academic research into their effectiveness in advertising; and the scarce research that has been undertaken has used self-reported measures of consumers' responses (Feng et al., 2019; Oh et al., 2020).

The features of 360-degree videos can drive new research that might capture the dynamic view and emotional responses they evoke by using continuous implicit measures. The literature shows that unconscious responses and emotions, measured through implicit metrics, influence decision-making (Eijlers et al., 2019). Unlike explicit responses, which are associated with conscious thoughts and emotions that can be assessed through self-report measures, implicit responses are associated with subconscious, automatic, and moment-to-moment reactions that lie outside the individual's awareness. Implicit responses are highly influential in decision-making processes (Khushaba et al., 2013). As Li (2019) pointed out, a high proportion of consumer responses are unconscious and intuitive. Thus, there is a need to assess the emotional and unconscious responses to advertising (for details, see Pozharliev et al., 2017). Furthermore, neuroscientific-based methods measure response changes in subjects on a continuous basis and are not, thus, affected by *post-hoc* reflection (McDuff, 2017). Eye-tracking (ET) has been extensively used as a tool for measuring visual attention in advertising (for a review, see Pieters and Wedel, 2004). The nature of 360-degree videos, which provide different viewing angles, makes gaze behavior (e.g., eye movements and fixations) an appropriate metric for measuring the visual attention paid to ad content (Jacob and Karn, 2003). Greater fixation time is associated with more detailed processing, and a lower number of fixations with poorer processing (Zhang and Yuan, 2018). Frontal asymmetry (FAA) is one of the most popular electroencephalography (EEG) metrics used to measure consumer choice (Telpaz et al., 2015). Based on the alpha band, right or left asymmetry is related to approach/withdrawal behaviors (Davidson, 1993, 2004). In this paper we refer to approach-related tendencies (or left-hemispheric dominance) as “positive emotional reactions,” and withdrawal-related tendencies (or right-hemisphere dominance) as “negative emotional reactions” (for a technical review, see Harmon-Jones et al., 2010; Fischer et al., 2018). Electrodermal activity (EDA) measures the electrical conductance of the skin based on its moisture (i.e., sweat) levels. An increase in conductance shows physiological activation (for a review, see Caruelle et al., 2019). Facial coding (FC) measures emotions through observing human facial muscle movements (McDuff, 2017). FC uses an objective-coding scheme, the Facial Action Coding System (FACS) (Ekman and Friesen, 1978), which describes facial muscle movements and the emotions related to the movements. Despite the advantages of neuroscientific measures, they have been very little used in advertising research (Chang, 2017). 360-degree video ads can help advertisers create emotional connections with their customers, and elicit neurophysiological responses that can be used to measure their effects.

No previous consumer neuroscience studies have compared 360° and 2D ads. Therefore, this study compares cognitive and emotional responses to 360-degree and static videos watched on PC screens; the neurophysiological metrics are based on electroencephalography eye-tracking, facial-coding and electrodermal activity. This study differs from others reported in the previous literature in: (i) the type of responses measured, that is, unconscious measures; (ii) the scope of the analysis is a continuous measure of the unconscious responses evoked by, and attention paid to, specific elements of ads.

Based on the interactivity and cognitive overload literature, and on the motivation, opportunity, and ability theoretical frameworks, and the advertising response model (ARM), we compare the effects of existing dyads of 360-degree and standard video ads on consumers' emotions and cognition. The research goal is 2-fold. First, to measure the effectiveness of 360-degree video ads vs. standard format video ads based on visual attention paid to specific elements of the ads, such as the embedded brand logo, and the emotional connectivity developed between the consumer and the brand. Second, to analyze whether interactivity and users' control result in higher levels of engagement in the viewer's prefrontal cortex.

Through this research we provide a theoretically grounded explanation for the persuasive power of 360-degree ads. The study contributes significantly to the knowledge of how consumers process interactive advertising. First, we explain the information processing of online video ads by assessing the influence of interactivity on the visual attention paid by the viewer to the ad, and on central (brand logo recognition) and peripheral information processing (emotions and asymmetry). Second, using unconscious measures, the present study is the first to explain the mechanism through which 360-degree video ads outperform standard versions.

## Conceptual Framework

### Interactivity in 360-Degree Video Ads

With its ability to give the user the control to manipulate his/her point of view, a 360-degree video provides two-way communication between the viewer and the message/interface and touches on a core concept in communication technology, that is, interactivity (Macias, 2003; Sundar et al., 2014; Feng, 2018). The ability to interactively change one's viewpoint resembles real-world navigation, which enhances the customer experience and increases emotional responses.

In a 360-degree format, in contrast to the traditional video format where the point of view is determined by the creative director, and viewers are passive, viewers have a free and omnidirectional viewpoint which they can move arbitrarily through all the angles of a 360-degree radius. Thus, consumers have the freedom to explore ad content based on their interests, and are not restricted by the creator's or the director's choices, to “navigate” in real time through the video scenes (Wijnants et al., 2015), and to decide “where and what” to look at (Hsiao and Grauman, 2017). That is, consumers are able to control, customize, and change their viewing experience and information flow. Active control, as a dimension of interactivity, has been described as a voluntary, instrumental action that directly influences the controller's experience (Liu and Shrum, 2002). Previous evidence suggests that, overall, viewers prefer dynamic videos (e.g., 360-degree) over 2D, static view videos (Broeck et al., 2017; Feng et al., 2019).

Interactivity in this context may result in users paying different levels of attention and varied amounts of cognitive workload (Feng, 2018). Consumer interaction with online ads can be categorized into a hierarchy of stages: pre-attention, attention, and behavioral decision (Chatterjee, 2001). A successful ad must initially grab the consumer's attention and then elicit emotions and induce recall. The multiple views provided by 360-degree videos make it necessary to address their effectiveness on a continuous basis to capture these dynamics.

### Visual Attention and Persuasion in Advertising

Attention has been recognized as the primary factor in advertising effectiveness since the appearance of the earliest models, such as AIDA (Edward, 1925). Without attention, advertising cannot persuade the consumer (Edward, 1925; Cao, 1999). The advertising response model (ARM) provides a framework for evaluating advertising performance; it integrates several measures and explains that winning the customer's attention is the most important feature in advertising (Wells and Loudder, 1997). Two alternative persuasion routes are described in the ARM, the central and the peripheral. During central processing the focus is on the product/brand, whereas during peripheral processing the creative executional aspects of the advertising are dominant. Both central and peripheral processing lead to ad liking and, ultimately, to purchase intention. Both routes were adopted also in the earlier formulated elaboration likelihood model (ELM) (Petty et al., 1983). Both processing routes are influenced by involvement levels. Under high involvement, recipients process information via the central route using a high level of brand-related message elaboration. Peripheral processing occurs under low-involvement conditions, and subjects typically rely on available peripheral cues, such as music, the source, or the spokesperson.

The advertising literature suggests that the motivation, opportunity, and ability (MOA) to process information are key factors in advertising content processing (MacInnis and Jaworski, 1989). *Motivation* relates to areas of the consumer's interest that condition his/her *opportunity* to view some parts of the ad, and *ability* relates to the users' competence in navigating in 360-degree videos. Therefore, the MOA approach provides a good explanation for the differential responses to 360-degree and standard videos. The attention capture and transfer model by elements of advertisements (Pieters and Wedel, 2004) describes a top-down (person and process) and bottom-up (stimulus) mechanism of the visual attention paid to brands, pictorial content, and text in print advertisements. Extending this model to 360-degree videos it can be argued that bottom-up factors (e.g., the angle of view of the ads) determine the level of attention paid. Furthermore, the top-down factors, which are driven by the viewer's motivation and interest, are in line with the motivation and ability variables of the MOA model, and also determine attention.

### Central Processing Route of Advertising

The advertised brand is widely recognized as one of the relevant cues in any ad (Geuens and De Pelsmacker, 2017; Belch and Belch, 2018). Attention and memory are the processes through which viewers become aware of, encode, store, and retrieve information. Thus, brand awareness plays a key role in advertising effectiveness. Brand awareness has been described as the recognition, or memory, of a brand (Huang and Sarigöllü, 2012). In 360-degree videos an interesting research question is whether the consumer's interactivity with video ads impacts on his/her processing of brand logos embedded in online video ads. As previously mentioned, in traditional ads the advertiser drives, and schedules, attention flow. However, in 360-degree ads the viewer selects the focal points and might avoid, even unintentionally, some parts of the ad, such as the brand name or logo. Accordingly, and irrespective of the consumer's interest in the ad, his/her exposure to the brand might be influenced by the reduced attention (s)he might pay to the brand. Following the MOA approach, certainly, if consumers are motivated by the brand as a key communication element, their attention will be high in either format (e.g., traditional and 360-degree). However, an interactive format may lead to less attention being paid to the brand logo because the angle of view adopted by consumers might reduce their opportunity to be exposed to some specific cues, such as the brand.

### Peripheral Processing Route of Advertising

In 360-degree ads consumers view some elements based on their personal choice. This active way of watching ads has been associated with the “functional view” of interactivity, as opposed to the “contingency view” as suggested by Sundar et al. (2003). Functional interactivity leads to higher peripheral processing (Sundar and Kim, 2005). On the basis of distinctiveness theory (Rosenkrans, 2009), it can be argued that motion supports salience because human beings prefer moving objects (Sundar and Kalyanaraman, 2004). Furthermore, in web ads, the dynamism conveyed by changes in ad content (e.g., animated ads) is more appealing than the static content in traditional ads (Sundar and Kim, 2005). Extending previous research to 360-degrees ads, the ad elements that viewers look at under their own control elicit positive attitudes. The limited capacity model (Lang, 2000) proposes that consumers encode, store, and memorize only a few information cues. This view suggests that the salience of information is determined by the consumer's goals or by the novelty and unexpectedness of the stimuli (Lang, 2000). The ARM suggests that the attitude formation process is shaped by the novelty of the interaction, which is dominant in peripheral processing.

### Contradictory Emotions and Processing Over Time

The advertising effectiveness literature discusses the positive influence of emotional responses on post–exposure attitude and recall (Holbrook and Batra, 1987; Morris et al., 2002). From the cognition perspective, emotions are mental states of readiness that arise from cognitive appraisals elicited by consumption; and they are accompanied by physiological processes (e.g., facial features) (Bagozzi et al., 1999, p. 184). The basic emotion approach (Plutchik, 1982; Li et al., 2015) proposes there are eight basic emotions innate to all humans (i.e., fear, anger, joy, sadness, disgust, surprise, trust, and expectancy) and many secondary emotions derived from these primary emotions.

When consumers are exposed to continuous stimuli (e.g., online video ads), their information processing might evolve over time from cognitive to emotional reactions, and from positive to negative encodings. Furthermore, Feng (2018) found that 360-degree videos evoked positive or negative emotions based on ease of navigation. In neuroscience studies these effects have been associated with the term “frontal brain asymmetry.” To explain frontal brain asymmetries in valence emotional processing, Davidson's model (1979) proposed that emotion-related lateralization is observed because emotions contain approach and/or withdrawal components. Therefore, emotion will be associated with right or left asymmetry to the extent to which it is accompanied by approach or withdrawal behavior (Davidson, 1993, 2004). In the present study we refer to approach-related tendencies (or left-hemispheric dominance) as “positive emotional reactions,” and withdrawal-related tendencies (or right-hemisphere dominance) as “negative emotional reactions” (for a review, see: Fox, 1991; Davidson, 1993; Davidson and Rickman, 1999). Frontal brain asymmetry has been applied in consumer choice (Ravaja et al., 2013; Telpaz et al., 2015) and advertising studies (Ohme et al., 2009, 2010; Daugherty et al., 2016; Guixeres et al., 2017). In interactive environments, to measure moment-to-moment emotions the optimum approach is to undertake brain wave analyses (e.g., EEG) in parallel with eye-movement observations. This form of integration can enrich the understanding of what emotional reactions consumers experience when they view an advertisement (Ohme et al., 2011; Guo et al., 2018).

## Hypotheses Development

**Figure 1:**
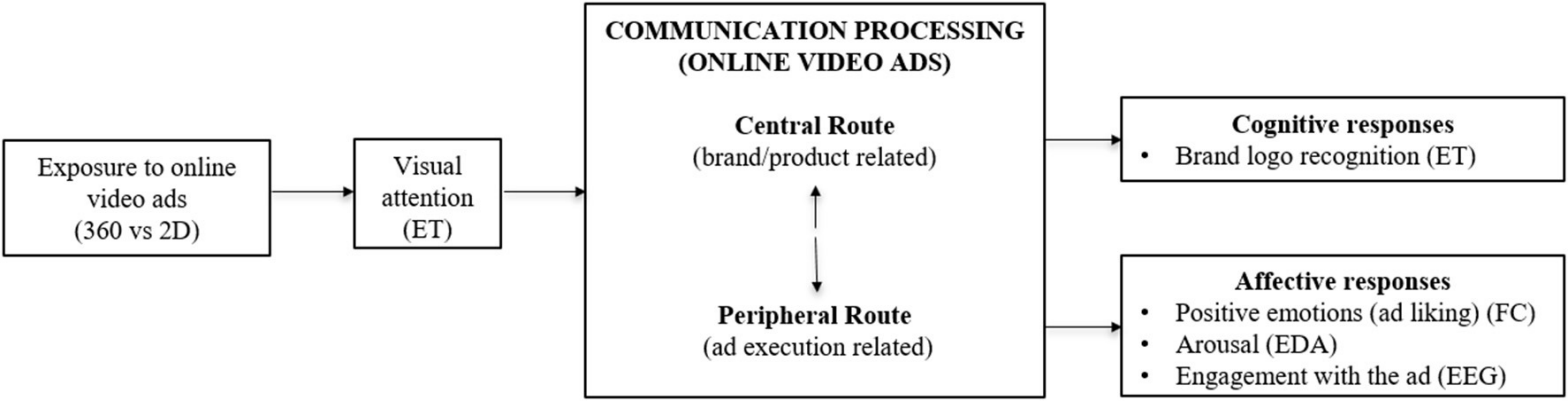
Conceptual framework scheme and neurophysiological measurements. ET, Eye-tracking; EEG, electroencephalography; EDA, Electrodermal activity; FC, Facial coding.

Grounded in the ARM model, this study posits that 360-degrees video ads have superior advertising effectiveness in terms of affective responses to the ad (engagement with the ad and intensity and arousal of the emotions evoked by the ad), and that 2D ads are more effective in terms of the cognitive processing of the brand logo. In this section, we discuss the expected relationships among the model variables and propose a set of hypotheses. Figure 1 shows the conceptual framework scheme and the neurophysiological tools used to measure the unconscious responses of consumers to online video ads with high/low interactivity.

### Interactive Advertising and Visual Attention

During 360-degree interactive ads the consumer has to focus his/her attention more and make higher cognitive processing effort than with traditional ads (Jung et al., 2014), which increases the use of his/her cognitive resources (Ariely, 2000; Tremayne and Dunwoody, 2001; Hupfer and Grey, 2005; Feng, 2018). Lang (2000) argued that people have limited information processing capacity. When an individual is faced with an overwhelming amount of information, (s)he needs to use a large amount of cognitive resource to process it. During these processes, if the individual's allocated cognitive resources fall short of the required level, cognitive overload can occur (Feng, 2018). In this regard, Hernández-Méndez and Muñoz-Leiva (2015) used eye-tracking to demonstrate that tourists' visual attention differed based on the degree of interactivity of online advertising. They found that, as animated banners require higher cognitive resources than static banners, less visual attention is paid to animated than to static banners (consumers fixate first on static banners, then on animated banners). Using FMRI, Oren et al. (2016) showed that performing a secondary task while viewing a video impaired viewers' memories of those videos. In the context of this study, given that the consumer decides the angle at which (s)he views the 360-degree ad, his/her visual attention may vary. Therefore, it is expected that higher interactive content will lead to higher cognitive processing levels. As curiosity stimulates interactivity, it leads to lower attention being paid to each piece of information. Interactivity is driven by the computer's mouse and, therefore, it requires more brain activity, which creates a negative impact on other physiological activities, such as viewing. Thus, the dynamic nature of 360-degrees ads means that fixation time, and the number of fixations, on specific parts of an ad will be lower in 360-degrees ads than in 2D-ads. Thus, we posit that the greater is the consumer's interactivity with video ads (360-degree vs. 2D), the lower will be the visual attention (s)he pays to the ad content.

**H1**. The visual attention paid to 360-degree video ad content is lower than the visual attention paid to 2D video ad content.

### Effects of Interactivity on Cognitive Processing: Brand Recognition

It is still unclear how 360-degree videos affect the processing of the different pieces of information embedded within ads. Of particular interest is the brand logo. Several explanations have been proposed in other contexts. First, previous research has demonstrated that television viewers have poorer recall of ad content in high-involvement program contexts, compared with low-involvement contexts (Coulter and Sewall, 1995; Coulter, 1998; Coulter and Punj, 1999). Pieters and Wedel (2004) found that the content of animated online ads is remembered less than the content of static online ads. Second, although a highly interactive ad helps immerse consumers in the brand story, this may cause cognitive overload (Feng, 2018). Pleyers and Vermeulen (2019), using eye-tracking, showed that the attention viewers pay to an ad, and ad effectiveness, can be impaired by interactivity and the control offered by interactive online media. Compared with the traditional television medium, memory for the ad is significantly reduced when it is shown with surrounding stimuli in interactive online media. These explanations fit well with the MOA and the ELM, as follows. The opportunity to watch a specific piece of information, such as the brand logo, is conditioned by navigation behavior, that is, the viewer can choose to look at it, or not. In 360-degrees video ads the least novel piece of information is the brand itself. Therefore, it is expected that consumers will look at it less or, at least, for a shorter period. Also, the brand is part of the central processing domain. As previously discussed, the novelty or unexpectedness of a stimulus relates to peripheral processing. Based on the above discussion, we argue that the greater is the consumer's interactivity with a video ad (360-degree vs. 2D), the less (s)he will look at the brand logo. Therefore,

**H2**. The visual attention paid to brand logos embedded in 360-degree video ads is lower than the visual attention paid to brand logos embedded in 2D video ads.

### Effects of Interactivity on Affective Processing: Engagement With the Ad

The digital communications' literature has shown that engagement with the media context increases advertising effectiveness (Calder et al., 2009). As Van Doorn et al. (2010) suggested, customer engagement behavior evolves over time; this perspective can be applied to 360-degree video ads because their content varies over time, albeit a short period of time. Therefore, a moment-to-moment measurement of the user's response (i.e., emotions) should be undertaken. The previous customer engagement literature is still inconclusive, depending on the conceptual framework used. In this study we adopt frontal alpha asymmetry (FAA), captured by the alpha wave of the EEG, that reflects engagement in paying attention to, or avoiding, an external stimulus (Clark et al., 2018; Ramsøy et al., 2018). Greater activation in the left or right brain hemispheres has been shown to indicate an approach toward the stimulus, and activation on the right shows avoidance (Harmon-Jones et al., 2010). At a cognitive level, the brain response literature (Berka et al., 2007) and advertising research (Ohme et al., 2010; Ravaja et al., 2013; Venkatraman et al., 2015) have proposed that frontal asymmetry is an indicator of user preference and engagement with advertisement content (Çakar and Gez, 2017). Thus, higher lateralization in the left part of the brain is an antecedent of positive connection with an ad (Ohme et al., 2011). Emotional connectedness is a process guided by dialogue, authentic connection, and relevance to the customer. Ohme et al. (2010) and Vecchiato et al. (2011) found that left hemisphere dominance was related to the pleasantness of TV commercial advertisements. Smith and Gevins (2004), using EEG, demonstrated that faster paced TV ads with frequent scene changes engaged viewers because they needed to continually redirect their gaze. In 360-degree video ads viewers can enjoy an engaging experience by deciding from moment-to-moment the angle from which to view the video scenes, and by arbitrarily moving their viewpoints to each one of the angles of a 360-degree radius. Customer participation, customization, and emotional connectedness are intrinsic to the 360-degree format, and have been identified as important drivers of customer engagement (Kumar and Pansari, 2016; Bleier et al., 2018). Thus, it is proposed that the more the consumer interacts with the video ad (360-degree vs. 2D), the greater will be his/her prefrontal cortex engagement with the ad.

**H3**: 360-degree video ads elicit higher frontal asymmetry than 2D video ads.

### Effects of Interactivity on Affective Processing: Emotions Evoked by the Ad

Emotions play an essential role in consumer behavior and, thus, understanding them is crucial for marketers (Laros and Steenkamp, 2005). Viewers usually experience a mixture of emotions (Hemenover and Schimmack, 2007). The literature argues that emotions are characterized by high excitement (Berger, 2011), such as joy and frustration (Gross and Levenson, 1995). Conversely, sadness or liking activate low excitation.

Previous studies have shown that viewer-ad interactivity evokes positive emotions in the viewers. For example, Horning (2017) examined the impact of second screen interactions on viewers' perceived enjoyment of news content. Oh et al. (2020) demonstrated that 360-degree videos had a greater indirect effect on the positive emotions evoked by video content, through enhanced perceived interactivity, than did 2D videos. Recent automatic facial expression detection-based studies measuring emotions evoked by ads have demonstrated that interactive advertising elicits positive emotions (e.g., Teixeira et al., 2012; Lewinski et al., 2014; Castellanos et al., 2018; Lacroix et al., 2020). Castellanos et al. (2018) used facial coding to compare the percentage of time viewers expressed joy when viewing interactive video ads and 2D video ads. Lacroix et al. (2020) used face reader facial expression recognition software to evidence that advertising perceived as highly experiential (highly interactive) produces more positive emotions (happiness) than advertising perceived as less experiential. Lewinski et al. (2014) used face reader to demonstrate that amusing video ads elicit greater happiness than non-amusing video ads. Teixeira et al. (2012) used facial coding to demonstrate that emotions such as joy and surprise can be leveraged to engage consumers watching Internet video ads. The higher interactivity and richer content of 360-degree video ads, indeed, may provide more amusement to consumers than 2D video ads, and therefore elicit positive emotions.

We expect that 360-degree videos will elicit positive emotions. The interactivity between a viewer and stimuli captivates the viewer and engenders positive moods through visual and auditory stimulation (Batat and Wohlfeil, 2009). Thus, it is proposed that the greater is consumers' interactivity with video ads (360-degree vs. 2D), the greater will be the intensity of their positive emotions.

**H4**. The positive emotions elicited by 360-degree video ads are more intense than the emotions elicited by 2D video ads.

A higher level of excitement leads to more effective message processing (Belanche et al., 2017). Previous research has suggested that interactivity affects involvement (Petty et al., 1983) and arousal (Fortin and Dholakia, 2005), which, in turn, elicit higher levels of attention and interest (Kensinger and Corkin, 2003). Bettiga et al. (2017) demonstrated, using EDA, that the higher is the consumer's interactivity with the product, the greater will be the influence of unconscious arousal on his/her attitude. Barreda-Ángeles et al. (2020) used EDA to evidence an increase in arousal associated with the immersive mode of viewing non-fictional videos. If we extend this reasoning to the context of this study, it is expected than the higher is consumers' interactivity with 360-degree video ads, the higher will be the arousal evoked by these ads, than that evoked by 2D video ads. Therefore, we propose the following hypothesis:

**H5**. The arousal evoked by 360-degree video ads is greater than the arousal evoked by 2D ads.

## Materials and Methods

### Sample

The study was conducted in the neuromarketing laboratory of a large European university. The sample consisted of 100 participants (47 females and 53 males, 19–67 years old, M = 43.09, SD = 6.49) recruited in the city where the laboratory is located. The participants were recruited by a professional market research company. The initial sample was reduced to 91 due to corruption in some of the acquired signals, that is, disconnection of a sensor during the test (3 subjects) and signals affected by artifacts (6 participants). The participants all had normal, or corrected-to–normal, vision and hearing. They were compensated for their participation with a gift card of 20. The study was approved by the ethical committee of the institutional review board of the university. Written informed consent was obtained from all subjects, in accordance with the Declaration of Helsinki.

### Experimental Design and Stimuli

A 2 factor (ad video format display: 360-degree vs. traditional ad) X 2 (product type: FMCG vs. durable product) between-subjects experimental design was implemented. Car and beverage brand ads were chosen because of their popularity and because one is a durable product and one a FMCG. The criteria for selecting the ads were: (i) to find two official advertisements for each brand in the two different formats (360-degree and 2D) (Table 1); (ii) two products within the same category were needed to control for brand influence and different market share levels, and to address brand familiarity.

**Table 1:** Ad description.

	**2D**	**360-degree**
Nescafe	https://youtu.be/VOOvpXFOPpY	https://www.youtube.com/watch?v=beo_Ln00RGc
Lipton	https://www.youtube.com/watch?v=7Myri_gIqT4andt=2s	https://www.youtube.com/watch?v=S_hpD7teoowandt=11s
Honda	https://vimeo.com/158687455	https://youtu.be/p3JKK32xkaU
BMW	https://www.youtube.com/watch?v=AfmMAVWK4Sw	https://www.youtube.com/watch?v=EEmuY2bC3SE

Based on these criteria the following four brands were chosen: BMW, Honda, Nescafe, and Lipton. BMW has 6 times the sales of Honda in the market under study (Statista, 2019). Nescafé is the leading instant coffee brand in Spain (5.9 million drinkers); Nestea is the leading iced tea brand in Spain (6.4 million drinkers), much more popular than Lipton iced tea (1.6 million drinkers) (Statista, 2019). Furthermore, per capita consumption of coffee is 3.8 times that of tea in the market under study (Ministerio de Agricultura, Pesca y Alimentación, 2019).

**Figure 2:**
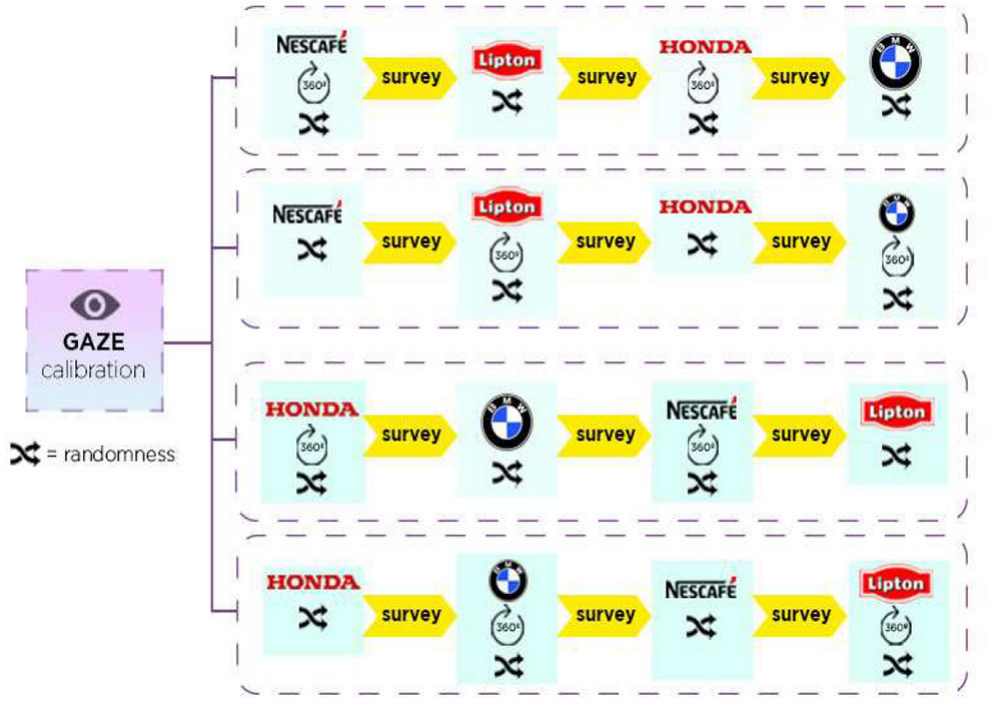
Stimuli and experimental design.

After the participants were welcomed into the laboratory, they were asked to complete an informed consent form and the study was explained. The EEG/EDA device was placed on the subjects while they listened to the explanation about the visual stimuli they were about to be shown. The ET and FC devices were embedded in the desk monitor. A specific baseline for EEG was used, which included a three-choice psychomotor vigilance task (3CVT), an eye-open task (EO), and an eye-closed task (EC), each of which were 3 min long. Next, the participants' brain and eye movements were calibrated. ET was calibrated through 9 points that appeared on the monitor. When the calibration was excellent, or good, the participants were randomly assigned (to avoid bias in the data gathering) to one of the four scenarios depicted in Figure 2. Depending on the scenario to which they were assigned, the participants viewed four video ads in the sequence shown in Figure 2. For example, participants in the first group were exposed to the ads as follows: (i) 360-degree video ad for Nescafé; (ii) 2D video for Lipton; (iii) 360-degree video ad for Honda; and (iv) a 2D video for BMW. The resolution of the ads was 1920 × 1080 pixels in both formats. The 360-degree format was viewed using Kolor 3.2. software that allows the users to interact with the videos; the quality is similar to YouTube videos.

After watching the videos the participants completed a short survey about their likeability for the ads (one item, five-point scale -“none” to “a lot”-), and their willingness to buy the product if money was available (1 item, 5 point scale –“definitively not” to “definitively yes”). Table 2 shows the behavioral measures of purchase intention and ad liking. The two ads most liked by the viewers were the 2D version of the Nescafe video ad and the 360-degree version of the Lipton video ad; no significant differences were found across the brands in each format.

**Table 2:** Behavioral measures.

**Brand**	**Variable**	**2D ads**		**360-degree ads**		***p*-value**
		**mean**	**S.D**.	**mean**	**S.D**.	
BMW	Purchase intention	2.96	0.87	2.94	0.94	0.929
	Ad liking	3.71	1.07	3.52	1.16	0.400
Honda	Purchase intention	2.50	0.85	2.25	0.86	0.147
	Ad liking	3.13	0.87	3.19	1.09	0.733
Nescafe	Purchase intention	3.08	0.84	3.08	0.85	0.969
	Ad liking	4.02	0.87	3.56	0.72	0.661
Lipton	Purchase intention	3.00	0.77	2.88	0.79	0.423
	Ad liking	3.65	0.99	4.10	1.09	0.599

### Measurement of the Neurophysiological Responses

#### Visual Attention: Eye-Tracking

Visual attention was analyzed using the Tobii Pro TX300 eye-tracking device. This records at 300 Hz and has a built-in 23-inch monitor. Following the recording of the gaze behavior, a speed-based fixation detection algorithm, with a threshold of 30 degrees/second, was used to identify the number of fixations and the average fixation time for each stimulus. To measure brand exposure, every second that the brand logo was shown in the advertisement was monitored through a dynamic AOI (area of interest) (Guixeres et al., 2017). We analyzed the percentage of visitors who fixated at least once on the AOI, the number of fixations, and the time spent viewing the brand-related AOI. The signals were synchronized with the stimuli through the iMotions Attention Tool (https://imotions.com/guides/).

#### Consumer Engagement With the Ad: Frontal Asymmetry

To compute the FAA an EEG spectral analysis was performed in each epoch using Welch's method, with 50% overlapping. In particular, we calculated the spectral power of the alpha band (8–12 Hz), and frontal asymmetry was calculated using the following formula:

$$\begin{array}{l} \left[Frontal\;asymetry=\log\left(F3_{alpha}\right)-\log\left(F4_{alpha}\right)\right] \end{array}$$

Electrical brain activity was recorded using the B-Alert X10 device (Advanced Brain Monitoring, Inc., USA). This records in nine channels, located in the frontal (Fz, F3, and F4), central (Cz, C3, and C4), and parietal—occipital areas (POz, P3, and P4), using the international location system 10-20. The recording was made at 256 Hz. First, data from each electrode were analyzed to identify corrupted channels using the fourth standardized moment (kurtosis). If a channel presented more than 10% of flat signal the electrode was classified as corrupted. The EEG baseline was removed and a bass pass filter between 0.5 and 40 Hz was applied. The signal was segmented into epochs of one second, and an intra-channel kurtosis level of each epoch was used to reject the epochs with high noise levels. To detect artifacts caused by eye movements, blinking, and muscular activation, independent component analysis (ICA) (Gao et al., 2010) was applied; a trained expert manually analyzed all the components, rejecting those caused by artifacts.

#### Emotions: Electrodermal Activity and Facial Coding

The consumers' emotions were measured using EDA and FC. The EDA signal was measured using the Shimmer 3 device. The signal was pre-processed in two phases. First, the signal was down sampled to 10 Hz (Lang, 2000). Second, in the re-sampled signal, the artifacts were visually diagnosed and corrected using Ledalab (v.3.4.8,www.ledalab.de) via Matlab (v.2016a; www.mathworks.com). The EDA was measured in micro-Siemens, the number of peaks and their amplitudes being identified during each stimulus. These measures are correlated with an increase in the subject's arousal caused by external stimuli (Bach et al., 2010).

The FC was assessed with a Logitech QuickCam Pro 900 webcam (1,600 × 1,200, 30 fps) using the Emotient FACET library (Stöckli et al., 2018). This is based on the Facial Action Coding System, and it recognizes the probability from 0 to 1 that a subject is experiencing a specific emotion. It offers a set of seven basic emotions (joy, anger, surprise, fear, contempt, disgust, sadness). For each stimulus, we calculated the percentage of time that a subject was experiencing a specific emotion, using a threshold of 0.75.

### Data Analysis

The Gaussianity of the data was checked using the Kolmogorov-Smirnov test (*p* > 0.05, with null hypothesis of having a Gaussian sample). To explore the differences between the subjects' responses, in both the 2D and 360-degree formats, we carried out an unrelated two-tailed *t*-test. The data analysis was performed using Matlab 2018b. To simultaneously compare the four ads a Bonferroni correction was applied, decreasing the threshold to consider a test significant to *p* < 0.0125.

## Results

**Figure 3:**
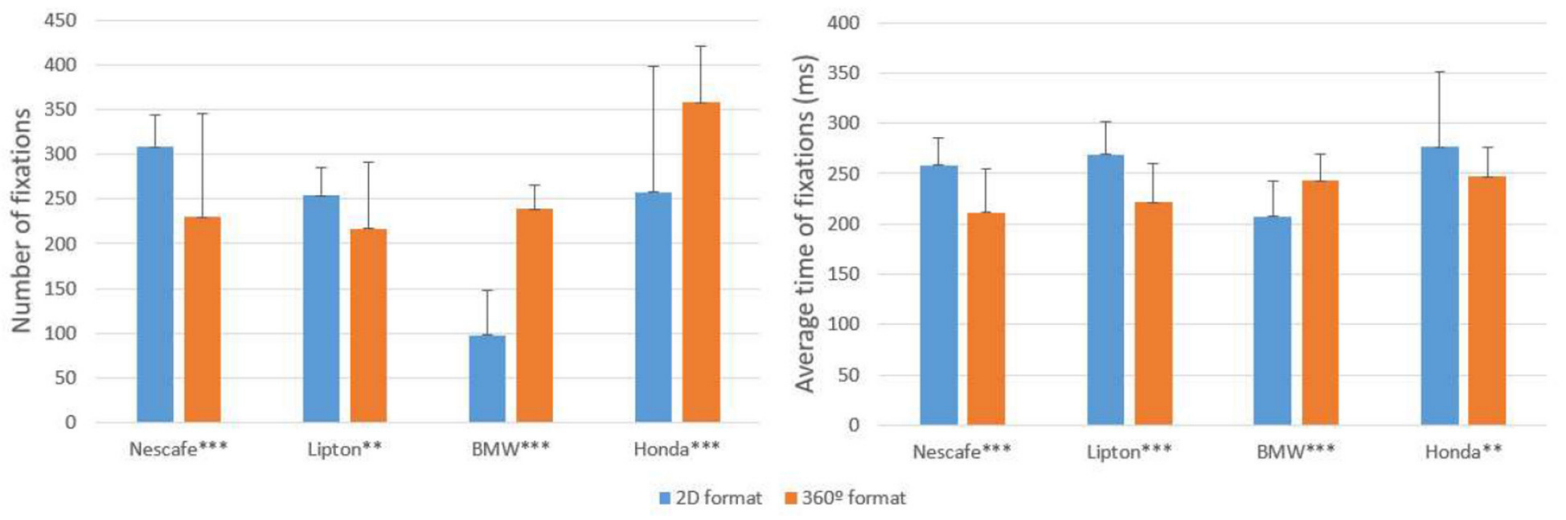
Visual attention paid to the ads. Values with significant differences: **p < 0.0125; ***p < 0.001. Bars represent the means, vertical lines represent the standard deviation of the means.

H1 predicts that the visual attention paid to a 360-degree video ad is lower than the visual attention paid to a 2D video. As Figure 3 shows, the number of fixations was higher in FMCG 2D ads than in FMCG 360-degrees ads. Furthermore, the average fixation time was greater in three out of the four ads. Overall, the participants paid more attention (longer fixation time) to the 2D video ads, except for BMW, than to the 360-degree ads. Therefore, H1 is supported for FMCG, but not for durables. It seems that the 360-degree format elicits a more dispersed view pattern that ultimately leads to a smaller number of fixations (except for durables with a high degree of narrative structure) and less average fixation time.

The higher number of fixations for the durable products can be attributed to the greater attention needed to choose a durable, based on their multiple product attributes, the narrative structure of the ad, and the complexity of the task to be performed in the car ad. The FMCG ads analyzed had a low, or moderate, level of narrative structure (Feng et al., 2019), with no clear plot connections between the scenes. Lipton's “Magnificent Matcha Tea” transports the viewer to a world of flavor represented by a series of exotic scenes (e.g., a woman stands in front of cherry blossoms outside a temple, a woman practices yoga beside the sea, a woman holds a cup of tea she intends to drink), and the Nescafé ad presents multiple scenes simultaneously featuring people from different countries at their breakfast tables drinking cups of coffee, while the song “Don't Worry” by Madcon plays in the background. In contrast, in the BMW ad the viewers are immersed in a simulated ride where they have to keep sight of a fashion model (Gigi Howard) driving a car in a race with other BMW cars (high narrative structure and goal-directed task). A close look at the BMW ad shows that it demands a task be undertaken; “Can you keep your eyes on Gigi?” If the consumer wishes to perform the task it would not be logical for him/her to change the angle of view because some pieces of the ad content would be lost and, consequently, it would be harder to “keep your eyes on Gigi.” Therefore, in the 2D format it is expected that participants will make fewer fixations. These fixations would be focused only on Gigi's position, not the rest of the ad. Conversely, in the 360-degree video, the participants might be motivated to look at other parts of the ad. The interactive viewing experience and the control needed to manipulate the point of view requires higher visual attention to be paid to specific parts of the BMW ad in the 360-degree version, than is required in the 2D version, to perform the task.

**Figure 4:**
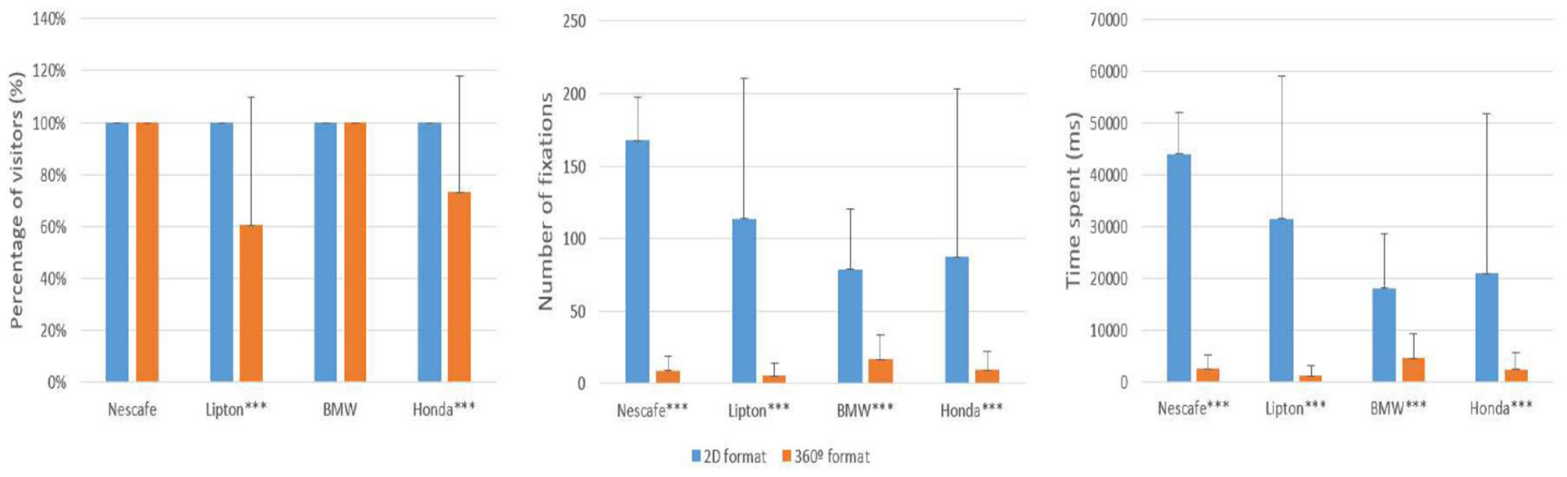
Visual attention paid to the brand logo. Values with significant differences: ***p < 0.001. Bars represent the means, vertical lines represent the standard deviation of the means.

H2 addresses brand recognition through their logos. Figure 4 shows the eye-tracking results for the brand logo area of interest in both the 2D and 360-degree video ads. Results are shown for the three metrics used: percentage of visitors, number of fixations, and time spent viewing the brand. The 2D stimuli attracted a higher percentage of visitors (*p* = 0.000), a higher number of fixations (*p* = 0.000), and more time spent (*p* = 0.013) viewing the brand, confirming H2. As expected, the visual attention paid to the brand logo is lower in the 360-degree video ads, because exposure to this specific element is influenced by the navigation paths adopted by the consumers. This finding is consistent with the MOA model and, more specifically, with the motivation and opportunity to process some parts of the ad. Furthermore, the areas viewed by the participants can be explained by the attention capture and transfer model (Pieters and Wedel, 2004), that is, they are based on a combination of personal factors and stimuli.

FAA was measured using EEG. The results of the brain responses comparing the 2D and 360-degree stimuli were analyzed using the mean and standard deviations of the asymmetry. The higher the FAA index, the higher is the approach behavior, with low values indicating withdrawal behavior. This metric has been related to the level of the engagement of the prefrontal cortex caused by content viewed (Ramsøy et al., 2018). Again, the consumers' unconscious responses produced an interesting result (see Table 3). The high interactivity of the 360-degree ads had a more positive impact on the engagement of the prefrontal cortex for high-involvement products (durables), than for low-involvement products (FMCG). However, the durables showed no significant differences in frontal asymmetry (H3 not supported). One possible explanation of the higher engagement of the prefrontal cortex in the participants who viewed the 2D version of the Nescafe ad, compared to those who viewed the 360-degree version, may be that the viewers of the 360-degree version suffered from cognitive overload. The ad simultaneously presents multiple scenes, featuring people from different countries at their breakfast tables, that don't follow any causal order. The multiple self-directed navigation possibilities offered to viewers by the 360-degree version may make it even more difficult to understand the chronology and causality of the narrative structure of the ad, leading to cognitive overload.

**Table 3:** Frontal asymmetry.

**Stimulus**	**Mean of frontal asymmetry**		
	**2D format**	**360-degree format**	***p*-value**
Nescafe	0.0259 (0.097)	−0.0302 (0.108)	0.0105^**^
Lipton	−0.00155 (0.102)	−0.0188 (0.113)	0.454
BMW	−0.0255 (0.122)	0.00854 (0.077)	0.121
Honda	−0.0347 (0.101)	0.0124 (0.084)	0.0206

Values with significant differences:

** *p < 0.0125. Frontal asymmetry is measured as the logarithm of alpha Power Spectral Density (μV2/Hz). Standard deviations are presented in parenthesis*.

H4 relates to the positive emotions elicited by the 360-degree and 2D ads; the FC results (depicted at Table 4) demonstrated that the 360-degree format provoked significantly high levels of joy and surprise, but only for the durable products. In the 360-degree ads the viewer is transported to vicariously share the story character's positive feelings and thoughts through an identification process which generates positive affect. The differences observed between the durables and the FMCGs can be attributed to the higher cognition needed for durables and to the focus of the ads. In the car ads the product (the car), is central, whereas the FMCG ads are based on fantasy. Unsurprisingly, it seems that ad content type may elicit different emotions. Moreover, and based on the differences between integral and incidental emotions proposed by Achar et al. (2016), the FMCG ads, which focus on fantasy, may be evoking incidental emotions, while the durable product ads, where the product is the focus, may be eliciting integral emotions. Therefore, H4 is partially confirmed.

**Table 4:** Positive emotions.

**Emotions**	**Stimuli**	**Mean of the percentage of time experiencing the emotion**		
		**2D format**	**360-degree format**	***p*-value**
Joy	Nescafe	38.35% (37.01%)	39.61% (33.86%)	0.870
	Lipton	20.64% (22.00%)	28.96% (29.53%)	0.146
	BMW	16.96% (19.92%)	44.57% (38.35%)	<0.001^***^
	Honda	21.78% (28.29%)	43.37% (34.60%)	<0.001^***^
Surprise	Nescafe	19.60% (21.63%)	15.51% (16.07%)	0.341
	Lipton	20.75% (23.47%)	21.71% (24.57%)	0.851
	BMW	12.38% (15.72%)	26.54% (23.72%)	<0.001^***^
	Honda	21.10% (21.13%)	33.99% (28.35%)	0.030

Values with significant differences:

*** *p < 0.001. Standard deviations are presented in parenthesis*.

**Figure 5:**
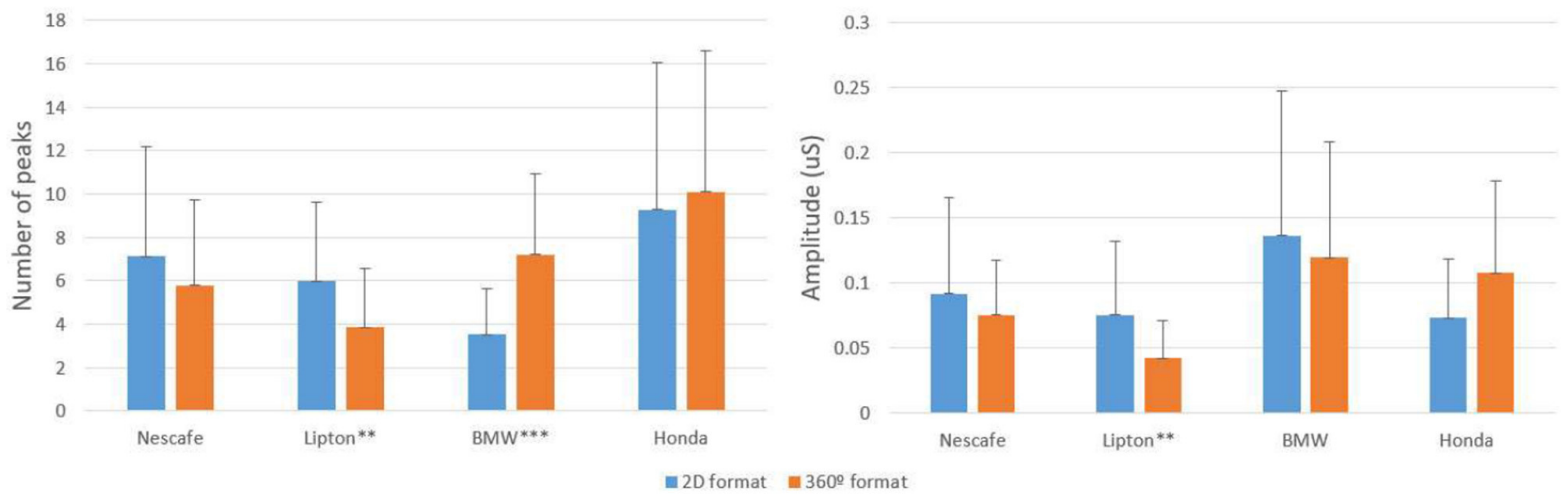
Number of peaks and amplitude of arousal. Values with significant differences: **p < 0.0125; ***p < 0.001. Bars represent the means, vertical lines represent the standard deviation of the means.

H5 predicted that 360-degree ads would elicit higher arousal; the EDA results comparing the 2D and 360-degree stimuli were analyzed using the mean and standard deviations of the number of peaks and their amplitude (Figure 5). Significant differences were found in the number of peaks, as follows. A greater number of arousal peaks were found in the FMCG ads with 2D formats than in the 360-degree versions, with the Lipton ad showing significant differences in the amplitude of its peaks. In the durable ads, on the other hand, more peaks were observed in the 360-degree ads. Therefore, H5 is supported for durables.

## Discussion, Conclusions, and Implications

The main goal of this study is to compare the effectiveness of traditional and 360-degree video ad formats through consumers' unconscious and continuous (e.g., moment-to-moment) responses to advertisements, obtained from EEG, ET, FC, and EDA.

The results showed significant differences in the visual attention paid, measured through ET, in the 360-degree vs. 2D ads. Indeed, the number of fixations and the time spent metrics were lower in the 360-degree videos than in the traditional, 2D videos. Therefore, 360-degree videos motivate viewers to watch specific parts of a video less than do 2D ads. This finding is consistent with the nature of 2D format ads. The 360-degree format allows viewers to look from multiple angles and, therefore, fixation can be dispersed, and the time spent on each stimulus will be lower. As previously stated, this conclusion is consistent with the MOA model and the top-down and bottom-up paths suggested by Pieters and Wedel (2004). Thus, motivation and top-down and bottom-up (i.e., stimuli) factors drive visual attention.

Although it is well-recognized that audiences actively participate in the online video advertising process by going beyond the messages presented in the videos themselves, and draw conclusions about brands, this inference process has received little attention in studies into interactive video ads. The application of the ARM model to online video ads provides insights that help to explain how online video advertising is actually processed, and to identify the strengths and weaknesses of 360-degree videos in comparison to standard versions. Brand recognition, measured through brand logo exposure and fixation time, was higher in the 2D ads. In other words, the gaze view directed toward the brand logo was higher in the 2D format than in the 360-degree format. Lower visual attention values are related to the viewer's freedom to explore content based on his/her interests (Su and Grauman, 2017). This finding is of importance for the design of brand communications as it is consistent with the MOA model. As in the 360-degree format the consumers' gaze cannot be directed, consumers will pay attention to stimuli based on their motivations. Therefore, exposure to the brand logo cannot be guaranteed as it is conditioned by consumers' opportunity to view the logo, and by their ability to handle the mouse to access different angles of view. In this vein, the present study advances our understanding of interactive advertising by examining cognitive overload from the perspectives of Lang's (2000) LC4MP and the interactivity literature. The results of this study challenge the proposal made in previous research that there is a positive relationship between degree of interactivity and ad persuasion effectiveness. Another interesting finding is that frontal asymmetry is higher in the 2D online FMCG video ads (Nescafe and Lipton) than in the 360-degree ads. Therefore, engagement with the ad depends on a combination of the interactivity of the ad and the cognitive overload caused by the ad.

This research assesses the influence of ad interactivity on emotions through facial recognition and EDA; 360-degree videos evoked more positive emotions, such as joy and surprise, than did traditional ads. As previously discussed, it is expected that greater interactivity will lead to more positive emotions (Rossiter and Bellman, 2005; Bellman et al., 2017). Our results also showed that the participants experienced joy and surprise for longer with the 360-degree ads. Moreover, the required active task in the 360-degree ad might have elicited higher viewer-brand interaction for the BMW ad which, in turn, might have evoked more positive emotions. In 360-degree ads viewers have to interact with the brands through the mouse; this evokes higher joy and surprise. We found that arousal, measured through number of peaks and EDA amplitude, was higher for FMCG 2D ads than for 360-degree ads. This unexpected result might have arisen because other variables, such as involvement with the ad or with the brand, are exerting a mediator effect. Also, in the 2D videos the creative director and producer decide the view sequence. If viewers change their point of view of the content, as they can in 360-degree videos, they can lose the thread of the story, which might cause lower levels of arousal. This result is quite close to the findings of Fortin and Dholakia (2005), who found no direct relationship between interactivity and arousal in a website setting. However, they found an indirect relationship mediated by involvement.

As regards the managerial implications, it is recommended that practitioners use 360-degree technologies to enhance positive emotions and customer engagement. However, the specific attention paid to the brand logo may vary depending on the viewing angle adopted by the users. The 360-degree video ads conveyed positive emotional responses, but these must be analyzed carefully throughout the entire ad. Thus, the 360-degree format is recommended under certain conditions. First, level of brand awareness must be high. This would mitigate the effect of the viewers not looking at particular stimuli, such as the brand logo, name, and other brand-related elements that may not be recognized by the consumer. Second, the length and content of the videos must be controlled to produce a balanced combination of joy and surprise. As 360-degree videos provide more emotional and immersive experiences for customers, they are powerful tools through which to create emotional connectedness. Third, advertisers of FMCG goods might use 2D online ads, which can cause higher prefrontal cortex engagement than 360-degree versions. 360-degree commercials need to be more detailed than 2D ads. Instead of looking at a scene from one angle, as in the 2D format, producers must take into account other angles, which will entail more sophisticated and expensive production. Four, advertisers need to make the effort to pre-test how ads are viewed, and from what angles. The challenge is to get the consumers to stick with the 360-degree video and keep them engaged.

From an academic viewpoint, this study makes the following contributions. First, it has been demonstrated that interactivity in video ads boosts positive emotions and enhances engagement. These two variables are commonly described as mediated objectives in advertising campaigns (Pavlou and Stewart, 2000). A strong relationship between interactivity, emotions, and prefrontal cortex engagement has been demonstrated in other digital formats, such as social media and websites. As Pavlou and Stewart (2000) noted, interactive advertising opens up new avenues for communication, but requires new measures of consumer responses (information search, visual attention, and ad information processing). Second, unconscious and continuous metrics based on neurophysiological tools are excellent means of accurately measuring advertising effects in interactive ads. Measures cannot be only explicit, they must also dynamically capture unconscious attention paid, and view angles. To the best of the authors' knowledge, the present study is first to assess 360-degree video ads using neurophysiological tools. Third, 360-degree ads represent customized visual communication driven by user participation. That is, they allow the viewers to take an active role that challenges the traditional assumptions about senders and receivers of advertising messages. This new view is in line with customer participation approaches.

Despite the benefits they provide in measuring continuous stimuli types, such as online videos, the physiological methods used in this study have some limitations and challenges. The study results have some limitations that can be sorted into two groups, the stimuli and the participants. First, our results derive from only four ads, and thus conclusions cannot be generalized to other types of ad, that is, with different creative content. Although the study controlled for brand influences and market share levels within the same category, we did not control the categories. The authors sought to analyze both well-known brands, such as Nescafe and BMW, and others with lower brand awareness in the market under study (Lipton and Honda). However, previous consumer experience with the brand might also bias the results of the study. Furthermore, participant-related factors, such as involvement with the brand, and with the ad, might also have exerted some influence. Another limitation is that we did not directly compare the preferences of the participants for the study stimuli. In future studies we will use a preference-based filter to better control this aspect.

Future research might expand in the following directions. First, the influence of content and executional factors, such as music and motion, might be considered. These executional factors might impact on emotions and cognitive workload. Second, the device used might have an impact. In this sense it would be interesting to analyze 360-degree videos viewed on mobile phones or on social media, and include cross-platform synergies, as suggested by Lim et al. (2015). Future research might also explore other 360-degree formats, such as 3D, and immersive settings based on augmented and virtual reality (Alcañiz et al., 2019; Wedel et al., 2020). This study measures engagement of the prefrontal cortex using frontal asymmetry; future studies might complement our findings by employing other measures of engagement. This work uses neuroscientific metrics (eye-tracking, FC, EDA, and EEG) to measure consumers' responses to advertising; to increase the robustness of the results obtained they might be compared to results of self-reported measures of the variables analyzed.

## Data Availability Statement

The raw data supporting the conclusions of this article will be made available by the authors, without undue reservation.

## Ethics Statement

The studies involving human participants were reviewed and approved by Local ethics committee of the Polytechnic University of Valencia. The patients/participants provided their written informed consent to participate in this study.

## Author Contributions

JA-A has developed the intellectual concept and experimentation part of the paper. MA has done part of the writing and planning of the experiment. JM-M together with JG have helped to collect and analyze the data. CR has helped the writing of the article and contribution in the experimental test. EB together with JA-A has helped to conceptualize the tasks and methodology of the experiment. All authors contributed to the article and approved the submitted version.

## Conflict of Interest

The authors declare that the research was conducted in the absence of any commercial or financial relationships that could be construed as a potential conflict of interest.
